# Formation and emergent dynamics of spatially organized microbial systems

**DOI:** 10.1098/rsfs.2022.0062

**Published:** 2023-02-10

**Authors:** Kelsey Cremin, Sarah J. N. Duxbury, Jerko Rosko, Orkun S. Soyer

**Affiliations:** School of Life Sciences, University of Warwick, Coventry CV4 7AL, UK

**Keywords:** microbial communities, microbial physiology, biogeochemical cycles, evolution, coexistence, system dynamics

## Abstract

Spatial organization is the norm rather than the exception in the microbial world. While the study of microbial physiology has been dominated by studies in well-mixed cultures, there is now increasing interest in understanding the role of spatial organization in microbial physiology, coexistence and evolution. Where studied, spatial organization has been shown to influence all three of these aspects. In this mini review and perspective article, we emphasize that the dynamics within spatially organized microbial systems (SOMS) are governed by feedbacks between local physico-chemical conditions, cell physiology and movement, and evolution. These feedbacks can give rise to emergent dynamics, which need to be studied through a combination of spatio-temporal measurements and mathematical models. We highlight the initial formation of SOMS and their emergent dynamics as two open areas of investigation for future studies. These studies will benefit from the development of model systems that can mimic natural ones in terms of species composition and spatial structure.

## 1. Introduction

In nature, microbes readily form physical structures such as biofilms, mats or granules [1–3]. Among these structures, biofilms are sometimes referred to as ‘self-organized systems' [4,5], but we note that any spatially organized structure can emerge through a combination of settling and attachment of cells on structured surfaces, and through the active chemical and physical action of microbes themselves, via processes such as motility, secretion of polymers and aggregate formation. Thus, we here refer broadly to microbial systems with any type of spatial structure as spatially organized microbial systems (SOMS). Self-organization and emergent dynamics can be involved in the formation and subsequent maturation of SOMS as we discuss further below.

SOMS form in a variety of environments ranging from the open ocean, freshwater and soil, to human and animal guts and lungs. In aquatic environments, SOMS readily form on sinking organic particulates, often termed ‘marine snow’, and result in metabolic activities that underpin biogeochemical cycles [6,7]. In SOMS associated with animal guts and plant roots, the dynamics of SOMS are believed to intertwine with host health [8,9]. Understanding metabolic activities and population dynamics within SOMS is, therefore, not only important for microbial ecology and physiology, but also of high relevance for studies focusing on the environment, animal physiology and host health. While the study of SOMS was at the origin of the field of environmental microbiology [10], the development of microbial physiology and molecular biology has been dominated by studies in well-mixed liquid cultures. In stark contrast to SOMS, well-mixed systems are characterized by all cells having equal access to media that they reside in, making experimental measurements and mathematical modelling easier, but resulting in a less representative, natural state for most microbes.

We have been seeing a return to SOMS in the last decade, with an increasing number of studies focusing on SOMS in the context of several central questions in biology [1,7,9,11–13]. What is the impact of spatial organization on cell physiology, compared to a well-mixed system? How does space shape species–species interactions on the competition–cooperation spectrum? What is the role of spatial organization in the emergence and maintenance of species diversity? Some important insights have been gained, towards answering such questions, from the study of simple SOMS. For example, studies on single-species biofilms—possibly the simplest form of spatial organization—have found that chemical and metabolic gradients form readily across the biofilm (reviewed in [11]), and influence individual cell metabolism to result in phenotypic divergence in local patches [14–20]. In the case of two or three species systems, the extent of local mixing, through an effect on local gradients, is found to influence the emergence and stability of species coexistence and metabolic interactions [21,22]. When interactions involve secreted proteins, such as scavenging enzymes, the stability of these interactions is also found to be affected by spatial organization [23–25]. In this case, a role for spatial sorting and mixing, arising from differences in growth rates of the interacting species or strains, is shown to be important (e.g. [26], and reviewed in [27]). Finally, the movement and mechanical interactions of cells in a biofilm can result in ‘self-organization’, giving rise to local or global aggregation patterns, or collective motility (e.g. swarming) [5,28–31].

In summary, in all SOMS evaluated to date, spatial organization is always found to have an impact on microbial system dynamics. Many questions remain unanswered, however, and there is still much more to learn from the study of different types of SOMS. In this perspective and mini review article, we focus particularly on the question of how spatial organization initially arises and develops in multi-species systems, and how it can impact subsequent species composition, diversity and evolution in those systems. For the latter question, we particularly highlight the capacity of spatial structure to provide a means for *emergent dynamics*. We note that, while the term ‘self-organization’ has been used before to describe swarming, emerging from motility and chemical or hydrodynamic interactions [28], the use of emergent dynamics should capture a broader range of possible behaviours arising from feedbacks between cell movement and physiology, evolution and environmental gradients (similar to the discussion in [31]). In the microbial world, these feedbacks can be intertwined due to short doubling times. Together, their feedbacks can impact spatial organization, stability, behaviour and function in a microbial community in unexpected ways.

## 2. Formation of SOMS—imposed versus emerged

How does spatial structure arise in microbial systems in the first place? The answer will probably depend on the system under study, but we can distinguish two broad routes. In one route, the system has an initial structure imposed externally. For example, many environments including marine snow, soil, animal guts and aquatic sediments already offer nutrient-rich, structured surfaces for microbial colonization. In some of these environments, there are additional, abiotically or biotically imposed chemical gradients around, or on, these surfaces (e.g. methane seepages [32], tidal sediments [3] and the gut [9]). The microbial colonization of these environments directly leads to structured microbial systems from the onset, initially through cell attachment, and then formation of biofilms on surfaces. An initial colony can then develop further into a so-called layered, microbial mat under some circumstances [3]. While mature microbial mats have been extensively studied [3], formation dynamics of mats are still not fully understood, and only a few studies have attempted longitudinal analyses of mat maturation [33].

New experimental approaches are slowly deciphering the colonization dynamics of nutrient-rich surfaces. For example, colonization of marine snow is mimicked with the use of magnetic beads that are covered with nutritious substrate and that can be readily extracted from aqueous media [34,35]. This has revealed stochastic effects on population dynamics during the early stages of colonization, arising from both the attachment process [34] and viral lysis [35]. In terms of attachment success, theoretical studies suggest that motility can be a key factor in colonization of surfaces in aquatic environments, as motile bacteria are expected to have two to four orders of magnitude higher diffusion coefficients than non-motile cells, and therefore increased particle encounter rates [36]. Recent experimental studies in milli-fluidic devices using wild-type, motile but non-chemotactic, and non-motile mutant cells of a chemotactic model organism show that the former can colonize sparsely distributed nutritious particles first [37].

**Figure 1.**
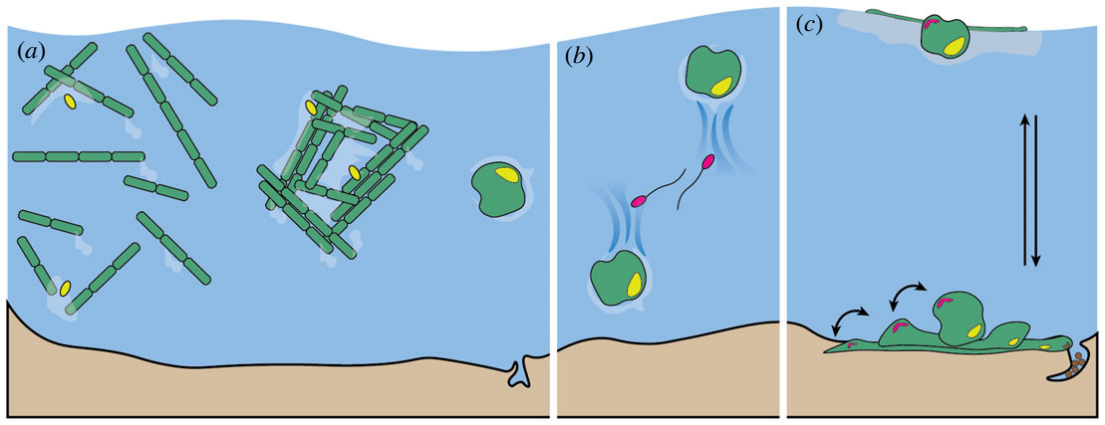
Formation of SOMS in an aqueous environment. (*a*) Schematic (not to scale) of the process of formation of photogranules. Filamentous cyanobacteria gradually aggregate and pack tightly into a granule. They are aided by secreted exopolysaccharides (light blue shading in *a*) and may sequester other planktonic microorganisms they encounter. (*b*) Photogranules may float or sink, depending on production and retention of gases within them. As they move, they may leave transient trails of chemicals and attract other microbial colonizers. (*c*) Cartoon representation of a speculative model, where granules may reversibly transition to and from the biofilm state, in response to changes in their environment or in the physiology of the constituent microbes. This process could take place either on a hard substrate or on the water surface, supported by an increased local viscosity due to the presence of secreted polysaccharides. Coupled with vertical motions from (*b*), this could enable entire communities to migrate towards favourable conditions.

In the second route to structure formation, the initial structure may itself result from microbial activity, such as motility-driven aggregation and secretion of extracellular polymers (figure 1*a*). This is a form of self-organization, whereby cell movement, attachment and interaction can lead to segregation, pattern formation or collective motion of cells with examples seen already in model systems of single-species biofilms [4,5,28–30]. In the case of multi-species natural communities, microbial aggregates and granules are found to emerge in aquatic environments, including the open ocean, and in bioreactors [2,6,38–44]. In some of these cases, the exact temporal dynamics and mechanistic processes leading to spatial organization are still unclear. In the case of phototrophic granules, the presence of motile, filamentous cyanobacteria has been highlighted as an important feature [2,39,42]. Some cyanobacteria are readily found to exist in aggregate colonies in nature [6,39,41], and can form aggregate structures under laboratory conditions [2,39]. Several cyanobacteria are known to secrete a range of extracellular polymers, often referred to as ‘mucus’, facilitating their gliding motility [45,46], and these can enhance aggregation. There might also be other physical factors affecting aggregation and structure formation, including hydrodynamic forces [40,44], as well as microbial traits. With regards to the latter, recent theoretical studies show that elongation, as seen in filamentous cyanobacteria, can increase the aggregation rate in turbulent aquatic systems [47].

It is, therefore, plausible that aqueous environments may readily result in physically driven initial aggregation of certain microorganisms (figure 1*a*), which can then lead to formation of larger granules through random colonization or recruitment of select bacteria from the environment (figure 1*b*) [2,6,39]. The granules may sink and become seed for a new biofilm [48]. However, depending on their community composition, they may be buoyant [49] and float to the surface, where a thin gelatinous layer made up of secreted polysaccharides [50] could provide enough structural support for biofilm growth, as seen before in liquid cultures of bacteria [51]. We speculate that the granule–biofilm transition is reversible and may enable whole SOMS to migrate in response to environmental changes (figure 1*c*).

Currently, it is not well understood if, and how, microbially driven structure formation in SOMS can result in selective enrichment of specific microbes in them. There is evidence that cyanobacterial and algal granules and aggregates in aqueous environments have a different microbial community composition than expected from a random assembly from the environment [39,52–58]. Interestingly, a similar effect is found with filamentous cyanobacteria dominating soil crusts in arid lands [59]. It is possible that such differences are due to selective enrichment of certain bacteria by algal or cyanobacterial secreted metabolites. A specific metabolic interaction of this type has been shown in one case [60], while broad induction of chemotaxis in response to cocktails of algal or cyanobacterial metabolic exudates is documented in several studies [60–64] (reviewed in [65]). There could also be selectivity in mucus and extracellular polymers secreted by cyanobacteria towards retaining certain bacteria. Such selectivity is implied in the case of corals and the animal gut, where control of mucus composition and properties is possibly used as a mechanism for enrichment of specific bacteria [9,66,67].

There is still a lot unknown about the exact physical processes and population dynamics of initial formation and subsequent development of SOMS. There are, however, emerging model systems from bioreactors [2,44,68], open ocean [69] and freshwater environments [39], which might allow these questions to be tractably addressed in the future.

## 3. From spatial gradients to feedbacks—emergent dynamics in SOMS

**Figure 2.**
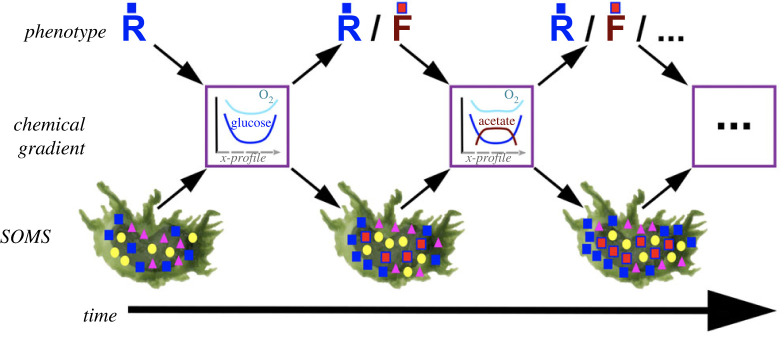
Illustration of the scales of dynamical changes and feedbacks in SOMS. This illustration depicts a section of a SOMS structure (bottom) that represents a sub-section of a larger three-dimensional structure that can grow over time. Different shapes represent different species, and the colour change of the squares represents different phenotypes. The spatial slice can be viewed from different perspectives, namely metabolic gradients and cell phenotypes. Metabolic gradients (middle) are depicted as concentration gradients along a cross-section of the SOMS patch by coloured traces on the drawn graphs. They can form due to local activity of cells, such as respiration of glucose. Cell phenotypes (top) are shown above, with two examples of metabolic phenotypes, respirers (R) and fermenters (F). The phenotypes can emerge due to local gradients and conditions, and can in turn lead to new gradients forming. This feedback between gradients and phenotypes can continue, and expand to different traits (as well as involve non-metabolic gradients, e.g. signalling molecules). Genotypic changes can subsequently follow on from these dynamics. See text for details.

We tend to regard microbial structures such as biofilms, mats and granules as static systems. However, the reality is that these systems harbour change at many levels and timescales (figure 2). At the most basic level, there are dynamic changes in metabolic and chemical gradients, as well as in cell positions due to migration, motility and growth. Closely interlinked with this, there is the possibility of phenotypic heterogeneity and switching at the cellular level [70]. This has been observed for metabolic phenotypes, in well-mixed cell cultures and within biofilms [15–17,20,71,72], as well as in other phenotypes such as polysaccharide formation and motility within biofilms [29,73,74]. Finally, at the level of the gross structure of the SOMS, there is dynamic change of the entire spatial structure—be it a biofilm or granule—due to new cells being recruited from the outside, to resident cells leaving or moving inside the system and to cell death and growth [25].

Among these dynamical layers (figure 2), metabolic gradients have been characterized in multi-species mats and granules [3,43,75]. In both cases, the gradients form either due to the overall structure of the SOMS, e.g. an inward-decreasing oxygen gradient forming on a granule due to consumption in the outer layers or limited diffusion, or within it, e.g. a local gradient forming due to some cells exhibiting fermentative metabolism (e.g. [14,20]). Both types of gradients are expected to be temporally and spatially dynamic (figure 2). Temporal dynamics can arise, i.e. gradients forming and disappearing, due to external conditions changing (e.g. light–dark, oxic–anoxic cycles [75]) or physiological changes within the resident cells of the SOMS. Spatial dynamics can arise, i.e. gradients can change direction or location, due to groups of cells moving within the SOMS. Movement can take different forms, including active motility with respect to existing gradients, such as light gradients (e.g. as seen in cyanobacterial diel movement in mats [76]), and passive movement due to being ‘carried’ by others [77], or being pushed and pulled due to growth and death-related physical forces [25].

We can imagine that the net effect of internal and external gradients forming, and species moving to the ‘tune of’ such gradients, would be a localization of each species inside their preferred domains within SOMS—as snapshots of spatial distribution in gut microbiota and environmental mats and granules suggest [9,33,75,78,79]. However, we argue that such static snapshots might be misleading and that we need to consider SOMS as environments of dynamic gradients that form and disappear both locally and across the entire structure. These dynamic gradients in turn are intertwined with the physiological responses of resident cells (e.g. [14–20,72]) and their movement.

The resulting picture is one of feedbacks: external and internal gradients influencing cell physiology and movement, and these in turn leading to consumption of existing gradients or formation of new gradients of metabolites, signalling molecules or excreted enzymes. These feedbacks are prone to creating nonlinear dynamics across time and space, including abrupt switching in system state (i.e. bistability) and emergent oscillations. Examples of switching of physiological states are well described in biofilms of yeast, where metabolic excretions can act as a threshold signal to cause glycolytic versus gluconeogenic metabolism [20]. Such metabolic switching of spatially distributed cells is also found to result in generation of additional metabolic gradients [72], in line with the dynamical and intertwined feedback view that we describe above (and in figure 2). In the case of biofilms involving motile species, the metabolic and signalling-based feedbacks on cell physiology are interlinked with the regulation of motility and with physical forces emerging from cell-to-cell interactions. The result of this increased complexity can give rise to further nonlinear spatio-temporal dynamics, such as pattern formation through cell aggregation, oscillations and travelling waves as seen in spatially structured colonies of the slime mould *Dictyostelium* [80–82] and in biofilms of several bacterial species [4,17,83–86]. In some cases, these dynamics seem to be primarily driven by swarming-mediated self-organization due to physical interactions [28], while in other cases they also involve secreted signalling molecules and enzymes [80–82]. The former cases are studied within the context of ‘physics of active matter’ [5], while the latter dynamics can be quantified and understood under the theory of ‘excitable media’ [87]. Both processes, however, are likely to be intertwined and indicated to have influences on biological functions, such as antibiotic resistance [15,18,88], division of labour [89] and development of complex structures and aggregates [80,82]. In each of these cases, there are intertwined dynamics of formation of metabolic or signalling gradients, influences on those gradients through cell responses and cell movement.

## 4. From short-term dynamics to evolution—the eco–evo feedback

The feedback dynamics that we described above, at the level of cell physiology and chemical gradients, can be intertwined with population growth dynamics and evolution within SOMS. In other words, SOMS can feature a further layer of feedback between microscale ecology, including cell physiology, and evolutionary dynamics: an eco–evo feedback (figure 2).

One clear example of how the eco–evo linkage can arise is the generation of ‘niches’. These are specific environments made up of biotic and abiotic conditions that influence growth rates of an individual or subsets of a community [90]. Dynamic nutrient, chemical or metabolic gradients within SOMS can present concentration ranges, which can be seen as multiple niches [11,91]. This can lead to genetic diversification of clones within biofilms due to local selective pressures [92,93]. As species change in abundance or metabolism, they can shift niches or create new ones, a process known as niche construction [90,91,94,95]. This process can allow new genotypes to successively evolve and subsequently coexist, as seen during long-term biofilm experimental evolution [91,96]. Morphotype succession and persistence can in this case occur due to successive niche construction, facilitated by cross-feeding of metabolites and spatially segregated surface adhesion. Evolution of niches and genetic diversification are, therefore, closely linked [90].

Another possible route for eco–evo coupling in SOMS is through spatial conditions impacting key evolutionary parameters within local patches. For example, it has been shown that stresses resulting from substrate limitation or generation of reactive oxygen species (ROS) can influence mutational patterns or rates, or rates of horizontal gene transmission [91,92,97]. Local stresses can readily arise within SOMS, thereby influencing mutational rates and processes within those local patches. For example, ROS gradients could be generated in phototrophic SOMS due to photosynthesis at high surface light or from rising oxygen levels and due to variable ROS scavenging capabilities within the structure [98,99].

Other key evolutionary parameters, such as fitness effects, genetic drift and strength of selection, can all be influenced by spatial organization. Selective forces overall are predicted to be weaker in SOMS due to population fragmentation [93], potentiating coexistence of variants with differing fitness levels. Additionally, there is experimental evidence for increased genetic drift and clonal interference in SOMS [91,100–102]. These effects can reduce competitive exclusion that is expected to dominate under well-mixed conditions, resulting in an increased diversity in SOMS. Indeed, it is shown that biofilms can fix a greater diversity of mutations and maintain such diversity for longer than in well-mixed environments [101]. A higher diversity is also found in spontaneous antibiotic resistant mutants in biofilms in the absence of antibiotics [103]. These same mutants were found to incur a fitness cost under well-mixed environments, indicating that they would have not been maintained as readily under those conditions as in biofilms [103]. Similarly, different bacterial species that normally exclude each other in liquid culture are found to coexist in biofilms and give rise to mutants that show high-yield phenotypes [104]. Other examples of physical segregation affecting evolutionary dynamics come from microbial systems with producer and cheater strains [23–25] and in the case of plasmid stability in biofilms [105]. Together, these findings indicate that SOMS, through physical segregation of cells, can reduce or replace competitive evolutionary dynamics that are expected to dominate under well-mixed conditions.

## 5. Conclusion and open challenges

**Figure 3.**
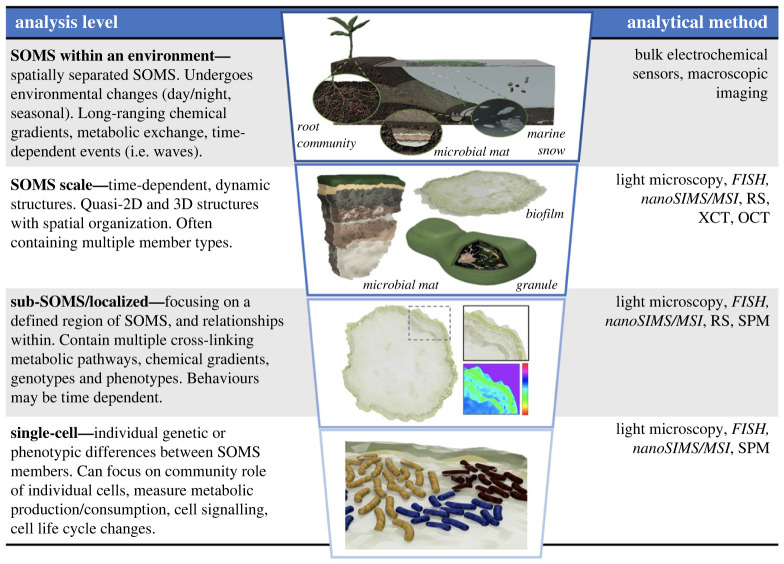
The different levels of analysis for SOMS. From top to bottom, analysis of SOMS together with their environmental parameters, SOMS-, sub-SOMS- and single-cell-level analyses. For each level, the possible analytical methods that can be applied are shown in the right-hand column. Methods in italics are those considered destructive, in that the samples would need to be fixed. Short notations and further details of methodologies are explained in box 1.

We argued here for the continued research of SOMS, with particular emphasis on their formation and on the emergent dynamics within them, at the levels of ecology, cell physiology and evolution. We hypothesize that emergent, nonlinear dynamics arising from ecology–cell physiology feedbacks are common to most SOMS and await discovery through application of appropriate measurements and models. Similarly, study of ecology–evolution feedback is so far explored in one- or two-species biofilms and synthetic systems [12,91,100–102], but should now move towards *in situ* measurements of natural or nature-derived SOMS. This shift in research focus will benefit crucially from development of tractable model systems and spatio-temporal measurement methods (figure 3). The former is now emerging in several contexts, as we highlighted above, while the latter are increasingly being developed (see box 1).

Box 1. **Measuring SOMS' dynamics in a temporal–spatial manner.**

Analytical techniques for studying SOMS should ideally quantify the localized, changing biological and chemical properties of interest. Here, we review several techniques for analysing SOMS and highlight possible areas for new methodological development. Figure 3 shows how these methods, some of which are covered in method-specific reviews (e.g. [106–109]), can be applied to different levels of examination of SOMS.

A powerful technique, that has a long history of application in SOMS, is the use of electrochemical probes to measure localized gradients [3,110,111]. Electrodes for detection of key metabolic species and processes are available, and their resolution can reach millimetre to micrometre scales. An expanding, exciting technique is scanning probe microscopy (SPM), which involves electrochemical probes with a nanoscale sensing tip, that are moved across a sample to map the surface. This can allow highly localized electrochemical measurements and have been used in mammalian systems to study respiration, local charge and membrane transport [112–114]. While SPM is only beginning to be applied to microbial cells [115,116], localized micro-electrode measurements have been combined with fluorescence *in situ* hybridization (FISH) (discussed below) in microbial biofilms to relate community structure to specific metabolic functions such as sulfate reduction and nitrification [79].

Live fluorescence microscopy imaging is another key technique that is increasingly used for investigating SOMS (e.g. [117–120]). Extrinsic fluorescent probes can serve as metabolite-, species- or cell-markers and can in some cases allow distinction of phenotypic variants (e.g. [71]). In the case of detection of metabolite concentrations, micrometre scale dye-containing beads have been developed (so-called optodes) [121,122]. Individual cell types or markers of cellular physiology can also be tracked using intrinsic signals (autofluorescence) [123–125]. Monitoring the dynamics of such intrinsic and extrinsic fluorescent probes via time-lapse microscopy can then allow observation of metabolic gradients and changes in cell location or physiology. Another approach is to genetically engineer fluorescent protein expression in cells of interest, allowing them to be tracked within biofilms or other SOMS [126].

In SOMS, spatial organization makes it difficult to detect or disentangle fluorescent signals. Applications of confocal, multi-photon—allowing deeper penetration—and light sheet microscopy to SOMS can in some cases allow spatial resolution (e.g. [109,127,128]), but further development in imaging technologies will be needed to fully address this challenge. In a recent example, several multi-photon imaging modalities were combined to analyse a soil microbe community at high resolution [129].

The dynamical changes in SOMS’ macroscopic structure might be driven by micrometre-scale local events, and it is currently difficult to simultaneously observe dynamics at both of these levels. Allowing a wide field of view for macroscopic imaging, while attaining high microscopic resolution, could allow new insights into SOMS. Emerging techniques in this direction include imaging platforms with specific optics [130,131], optical coherence tomography (OCT) [132] and X-ray computer tomography (XCT). All these techniques can be used in live cell imaging, including XCT; however, samples for XCT are often fixed or frozen for improved imaging stability and resolution [133]. As X-rays have deep penetration into a sample, they are used to image the sample from all directions, forming a series of two-dimensional (2D) radiographs which are then reconstructed to form a detailed three-dimensional (3D) projection of the sample [134]. XCT has previously been used to image biofilms in complex environments [135–137].

Another approach for imaging large structures is to fix the sample and then create micrometre-scale slices. This approach can still be used in a temporal fashion by using samples terminated at different time points in their ‘development’. Combining micro-slicing with FISH allows the ability to distinguish different species under a fluorescence microscope using custom-tagged, fluorescent nucleotide primers to target specific DNA or RNA sequences. Where novel microbial members may not have readily available FISH probes, recent developments have explored first performing a shotgun sequencing survey, and then targeting the most variable regions of the 16S rRNA gene sequence as FISH probes [138]. It is also possible to design FISH probes for a specific gene, thereby measuring the localization of a specific metabolic function of interest [139]. Recent studies combined FISH with micro-electrodes (as discussed above) and with micro-auto-radiography (MAR-FISH) [140]. In the latter case, MAR allows measuring radioisotope localization in samples that are incubated with a radiolabelled substrate that is exclusively involved in a specific metabolic process.

Imaging approaches, including FISH, can be paired with spectroscopic techniques such as Raman spectrometry (RS) and mass spectrometry (MS). The former can be applied to both live and fixed samples, while the latter has been primarily applied to fixed microbial samples so far (but a few examples of live microbial MS are also reported) [141,142]. RS measures the inelastic scattering of photons, relating to molecular rotation, and can detect many different types of molecules through their distinctive ‘Raman fingerprint’.

In complex biological systems, RS has the potential to identify thousands of individual molecules in a single scan, i.e. fingerprint; however, significant challenges lie in the deconvolution of these complicated signals. Thus, a key developmental challenge in the application of RS is to associate specific spectral peaks to their corresponding metabolites, in order to uncover the underlying biochemical or species-level functions. Biomolecules usually produce peaks between 600 and 1800 cm^−1^, forming a fingerprint for distinguishing species or phenotypes [143,144]. Chemometric methods can be used to interpret and extract chemical information from these spectra, with examples including principal component analysis and machine learning [143,145]. To date, current technology has been unable to achieve this level of analysis for full biological cell samples, indicating an area for development. Changes in Raman fingerprints between liquid or biofilm growth conditions have been observed [146], and different Raman fingerprints were observed across depth gradients in microbial mats [144,147]. Combining RS with FISH and confocal laser scanning microscopy showed that RS can be used to map species distribution in multi-species microbial communities and biofilms [148,149].

Mass spectrometry imaging (MSI) enables localized molecular analysis. The so-called nanoscale secondary ion mass spectrometry (nanoSIMS) is particularly useful in detecting different isotope forms of molecules due to the production of the secondary ions. It can, therefore, be used to track the assimilation of specific isotopes by SOMS members, identifying metabolic exchanges (e.g. [150]).

How SOMS form remains an interesting question from both biophysical and functional perspectives. While we have discussed some of the latest findings in the former category, the possible functional reasons for the formation of multi-species SOMS such as granules and mats remain understudied. One possibility is that SOMS formation in natural environments is a defence mechanism against grazing or harsh conditions, including viruses. There are only a few studies so far exploring the role of cyanobacterial granules as protection against grazing [151], but there is evidence for coevolutionary dynamics between grazers and granule-forming cyanobacteria [152]. In the case of biofilms, there are several studies showing biofilm formation affecting antibiotic efficacy and virus penetration [15,17,18,153,154]. A general protective effect for all SOMS and in different contexts, however, is yet to be established. A more generally applicable idea is that SOMS allow for more efficient energy dissipation, and arise in accordance with thermodynamic laws [79,155]. In support of this view, energetics of metabolic exchanges at thermodynamic limits—such as those based on hydrogen—are found to be improved by close proximity of cells in two-species biofilms [156]. To expand such findings to more complex multi-species SOMS requires their further study from an energetic perspective.

A well-established dynamical aspect in SOMS is the presence of metabolic and chemical gradients, as we highlighted above. How these gradients shift in time and space is still not well understood and requires integrated application of mathematical models and spatio-temporal measurements (figure 3 and box 1). The feedbacks between these gradient dynamics and cell physiology within SOMS can further feed into evolution, suggesting that SOMS might be the right level of study to understand proposed, fundamental trade-offs in cell physiology. For example, a trade-off between growth rate and motility has been demonstrated in *Escherichia coli* isolates from the gut environment [157], which is inherently spatial.

A highly exciting avenue of research is to try and connect environment–physiology–evolution feedbacks within SOMS to their higher-level function. To this end, some of the SOMS—such as phototrophic granules or mats—could be seen as emergent, self-sustaining ecosystems in their own right. Thus, SOMS might be ideal systems to study, for understanding closed, or semi-closed, microbial ecosystems [13,158]. For example, such systems can provide testing beds for the relevance, and possibility, of group selection in nature, a still debated topic [159,160]. Besides such fundamental studies on function, SOMS can also be exploited in a biotechnological context. For example, naturally emerging granules are being used in water treatment and bioproduction, and further understanding of these systems will enable directed engineering of their functions, as seen with phototrophic granules [161].

It is very tempting to view SOMS as early versions of multi-cellular organisms [1,3,11]. There is clearly the fact that SOMS comprise many cells in close proximity of each other, just as seen in tissues and organs. Beyond this clear parallel, however, there are a range of signalling and metabolic mechanisms in multi-cellular systems that are yet to be identified in SOMS. For example, the presence and possible role of mechanisms akin to programmed cell death in microbial systems are currently explored [162], while metabolic gradients in SOMS are likened to morphogen gradients [11]. The extent of these similarities, however, and any tissue-like behaviour in SOMS needs to be further elucidated. There is clearly more work to be done to understand environment–physiology dynamics and feedbacks in SOMS. As this work is undertaken, there would be immense benefit from development of more nature-like model systems and from interdisciplinary collaboration between developmental biologists, microbial ecologists/physiologists, biophysicists and mathematical modellers.

## Data Availability

This article has no additional data.
